# Nicotine Promotes Tumor Growth and Metastasis in Mouse Models of Lung Cancer

**DOI:** 10.1371/journal.pone.0007524

**Published:** 2009-10-20

**Authors:** Rebecca Davis, Wasia Rizwani, Sarmistha Banerjee, Michelle Kovacs, Eric Haura, Domenico Coppola, Srikumar Chellappan

**Affiliations:** Department of Oncologic Sciences, H. Lee Moffitt Cancer Center and Research Institute, Tampa, Florida, United States of America; Vanderbilt University, United States of America

## Abstract

**Background:**

Nicotine is the major addictive component of tobacco smoke. Although nicotine is generally thought to have limited ability to initiate cancer, it can induce cell proliferation and angiogenesis in a variety of systems. These properties might enable nicotine to facilitate the growth of tumors already initiated. Here we show that nicotine significantly promotes the progression and metastasis of tumors in mouse models of lung cancer. This effect was observed when nicotine was administered through intraperitoneal injections, or through over-the-counter transdermal patches.

**Methods and Findings:**

In the present study, Line1 mouse adenocarcinoma cells were implanted subcutaneously into syngenic BALB/c mice. Nicotine administration either by intraperitoneal (i.p.) injection or transdermal patches caused a remarkable increase in the size of implanted Line1 tumors. Once the tumors were surgically removed, nicotine treated mice had a markedly higher tumor recurrence (59.7%) as compared to the vehicle treated mice (19.5%). Nicotine also increased metastasis of dorsally implanted Line1 tumors to the lungs by 9 folds. These studies on transplanted tumors were extended to a mouse model where the tumors were induced by the tobacco carcinogen, NNK. Lung tumors were initiated in A/J mice by i.p. injection of NNK; administration of 1 mg/kg nicotine three times a week led to an increase in the size and the number of tumors formed in the lungs. In addition, nicotine significantly reduced the expression of epithelial markers, E-Cadherin and β-Catenin as well as the tight junction protein ZO-1; these tumors also showed an increased expression of the α_7_ nAChR subunit. We believe that exposure to nicotine either by tobacco smoke or nicotine supplements might facilitate increased tumor growth and metastasis.

**Conclusions:**

Our earlier results indicated that nicotine could induce invasion and epithelial-mesenchymal transition (EMT) in cultured lung, breast and pancreatic cancer cells. This study demonstrates for the first time that administration of nicotine either by i.p. injection or through over-the-counter dermal patches can promote tumor growth and metastasis in immunocompetent mice. These results suggest that while nicotine has only limited capacity to initiate tumor formation, it can facilitate the progression and metastasis of tumors pre-initiated by tobacco carcinogens.

## Introduction

Tobacco smoke contains a wide array of compounds that are deleterious to health; some of these compounds such as 4-(methylnitrosamino)-1-(3-pyridyl)-1-butanone (NNK) and N'-nitrosonornicotine (NNN) are nicotine derivatives and are highly carcinogenic [1]. These molecules can form adducts with cellular DNA, leading to mutations in vital genes like Ras, p53, and Rb [2], [3], [4]. While nicotine is the addictive component in cigarette smoke, it cannot initiate tumor formation in mice or rats, though it has been reported that it can initiate tumors in hamsters [5].

Nicotine exerts its cellular functions through nicotinic acetylcholine receptors (nAChRs), which are widespread in neurons and neuromuscular junctions [6], [7], [8]. nAChR subunits were found to be present in a wide variety of non-neuronal cells, including those of epithelial and endothelial origin [9]. nAChRs are pentameric proteins consisting of nine α subunits (α2–α10) and three β subunits (β2–β4) in neuronal cells [10] and can be grouped into two types- one comprising of a heteromeric pentamer of α2–α6 and β2–β4, and the other comprising of a homomeric pentamer of α7–α9 [11], [12], [13]. Non-neuronal (muscle type) receptors are composed of either α1, β1, δ and γ subunits in the embryonic form, or as α1, β1, δ and ε subunits in the adult form in a 2∶1∶1∶1 ratio [14]. Both muscle and neuronal type receptors are somewhat similar to one another, especially in the hydrophobic regions. They differ in their sensitivity to α-bungarotoxin (α-BT) [13], [15]. α7 nAChR is abundant in neuronal cells, has high permeability to Ca^2+^ thereby facilitating Ca^2+^-dependent events such as neurotransmitter release, regulation of second messenger cascades [16], [17], cell survival [18] and apoptosis [19]. The finding that nAChRs are present on non-neuronal cells was followed by the observation that nicotine could induce the proliferation of endothelial cells [20] as well as lung carcinoma cell lines in vitro and in vivo [21]. Nicotine has also been shown to protect lung cancer cells from apoptosis induced by standard chemotherapeutic drugs [22]; in addition, nicotine could induce proliferation through the mediation of β-arrestin-1, Src kinase and the induction of Rb-Raf-1 interaction [23], [24].

Recent studies from the Russo lab has shown that inhibition of nAChRs by α-cobratoxin can inhibit the growth of A549 tumors in immunocompromised mice [25]. Recent reports have also demonstrated the presence of functional estrogen receptors in lung cancer cell lines; estradiol has been shown to promote lung tumor cell proliferation [26], [27], [28], [29] and a combination of nicotine and estradiol can promote the growth of A549 tumors in athymic nude mice [30]. These reports present studies done on immunodeficient mice; we ventured to study the effect of nicotine on two separate models of immunocompetent mice. Studies presented here show that nicotine by itself can induce the growth and metastasis of tumors in immunocompetent mice, independent of other tobacco carcinogens. Nicotine administered either intraperitoneally or by commercially available transdermal patches could substantially promote tumor growth. Similar effects were observed on implanted tumors as well as tumors induced by the tobacco carcinogen, NNK. Further, mice exposed to nicotine showed significantly enhanced lung metastasis as well as tumor recurrence post surgical removal of the primary tumor. These results imply that nicotine can enhance the growth and metastasis of pre-established lung tumors.

## Methods

### Cell Culture and Proliferation assays

Line1 cells were cultured in RPMI (MediaTech) containing 10% FBS. Line1 cells were plated in poly-D-lysine coated chamber slides at a density of 10,000 cells per well and rendered quiescent by serum starvation for 72 h. Cells were then re-stimulated with nicotine for 18 h in the presence or absence of 10 µM α-BT, an α_7_ receptor antagonist. Cell proliferation was assessed by measuring BrdU incorporation. Bromodeoxyuridine (BrdU) labeling kits were obtained from Roche Biochemicals. S-phase cells were visualized by microscopy and quantitated by counting 3 fields of 100 in quadruplicate.

### Line1 Tumor Growth Experiments

Female BALB/c mice aged 26–30 days (Charles River) were clipped and depilated using Nair for hair removal on the back and flanks. Line1 cells (1×10^6^ per tumor) were harvested and resuspended in 100 µl of PBS for injection. The mice were randomized 3–7 days after injection of tumor cells. Mice were separated into two groups- Vehicle (*n* = 8) and Nicotine (*n* = 8) (patch or i.p. injection). Mice received nicotine by i.p. injection at a dose of 1 mg/kg three times a week. Nicotine was also applied using transdermal patches (Nico®Derm® CQ, GlaxoSmithKline) at a dose of 25 mg/kg daily. Patches (14 mg) were cut into 30 equal sized squares representing 0.45 mg of nicotine using a razor blade. The average weight of the mice was 0.018 Kg (18 g). A small square representing 0.45 mg of the patch equaled a final dose of 25 mg/kg daily. One patch was applied to the lower dorsal area (depilated) per day. Nicotine was administered for 2 weeks and tumor growth was measured thrice weekly. Nicotine levels in mice were analyzed using a cotinine ELISA kit (cat # CT086D; CalBiotech, Spring Valley, CA).

### Line1 metastasis experiments

Line1 cells (1×10^6^ per tumor) were injected and the mice were subsequently randomized into two groups. Group one received the vehicle (*n* = 16) and group two received nicotine (1 mg/kg) (*n* = 16) by i.p. injection thrice weekly. After 3 weeks of nicotine treatment, the tumors were removed under anesthesia and the skin was stapled, mice recovered on a warmed heating pad and the staples were removed after 7 days. Mice continued to receive nicotine or vehicle for an additional 2 weeks. At the end of the experiment, the mice were euthanized and the lungs were fixed in formalin.

### A/J tumorigenicity experiments

Two experiments were carried out using female A/J mice 4–6 weeks of age (Jackson Labs). The mice were maintained in accordance with Institutional Animal Care and Use Committee (IACUC) procedures and guidelines. NNK (NCI) (100 mg/kg) was administered to all mice (*n* = 16) once a week for 5 weeks [31], [32]. The mice were randomized into two groups; group one received the vehicle (PBS) (*n* = 8) and group two received nicotine (*n* = 8) by i.p. injection at a dose of 1 mg/kg three times a week for an additional 28 weeks. Nicotine levels in mice were analyzed using a cotinine ELISA kit. At the end of the experiment, the mice were euthanized and the lungs were fixed in 10% buffered formalin. The lungs were subsequently examined by stereoscope for number of lung tumors. The lungs were paraffin embedded and sectioned for IHC staining and pathological examination.

### Quantitation of Cotinine

Level of cotinine in urine was used as a marker for nicotine levels. Urine (100 µl) was collected throughout the length of the experiments and stored in −20°C for later analysis. Cotinine levels were determined by using the BioQuant Cotinine Direct ELISA kit following manufacturer's protocols.

### RT-PCR

Reverse transcriptase coupled PCR was done for α_7_ nAChR on Line1 cells. Total RNA was isolated from serum starved and nicotine stimulated Line1 cells (RNEasy Kit; Qiagen) by using the manufacturer's protocol. cDNA was synthesized by reverse transcription by using an AMV-RT kit (Promega). The primers and conditions for RT-PCR for α_7_ nAChR were described elsewhere [23], [33]. PCR for actin was used as the loading control.

### Noradrenaline and Adrenaline ELISAs

Line1 cells were rendered quiescent by serum starvation and treated with 1 µM nicotine for 48 h. After which, the media was collected and diluted appropriately to assess the noradrenaline and adrenaline secreted into the media. The ELISA was done using adrenaline research ELISA and noradrenaline research ELISA kits from Labor Diagnostika Nord (cat# BA 10–5100 and BA 10–5200 respectively) by following the manufacturer's protocol. The concentrations were reached at by following the calculations suggested in the protocol and the values are representative of two independent experiments.

### Immunohistochemistry

Upon termination of animal experiments, tumors were removed and fixed in 10% neutral-buffered formalin before processing into paraffin blocks. Tissue sections (5 µm thick) were cut from the blocks and stained either with H&E alone or with antibodies against α_7_ nAChR (1∶50 dilution, Abcam), E-Cadherin (1∶200 dilution, Santa Cruz), β-Catenin (1∶200 dilution, Santa Cruz) or ZO-1 (1∶200 dilution, Abcam). For immunohistochemical studies, paraffin sections were rehydrated to PBS and processed using the following protocol. Sections were rinsed in dH2O, and then subjected to microwave ‘antigen retrieval’ for 20 minutes on 70% power, with a 1 minute cooling period after every 5 minutes, in 0.01 M sodium citrate, pH 6.0. Sections were cooled for 20 minutes, rinsed 3 times in dH2O, twice in PBS and the rest of the staining was done following the manufacturer's protocol (Universal Elite ABC kit, Vector labs). For color development the slides were treated with peroxidase substrate kit from Vector labs (cat # SK-4100) and developed using DAB as chromogen. After a final rinse in dH2O, sections were lightly counterstained in hematoxylin, dehydrated and mounted with Clarion mounting medium (Santa Cruz Biotech.). Both H&E and immunohistochemically stained slides were scanned on an Ariol SL-50 (version 3.0.70) Automatic Scanning System from Applied Imaging. Tumor sections were scanned at 20×magnification and the regions of interest were identified and outlined by a pathologist (DC). The tumor sections with immunopositive regions from control and treated mice were quantified for intensity, size and area using Ariol software Review Station. p value was determined for statistical significance with the help of student's t-test.

## Results

### Nicotine promotes the growth of tumors in mice

**Figure 1:**
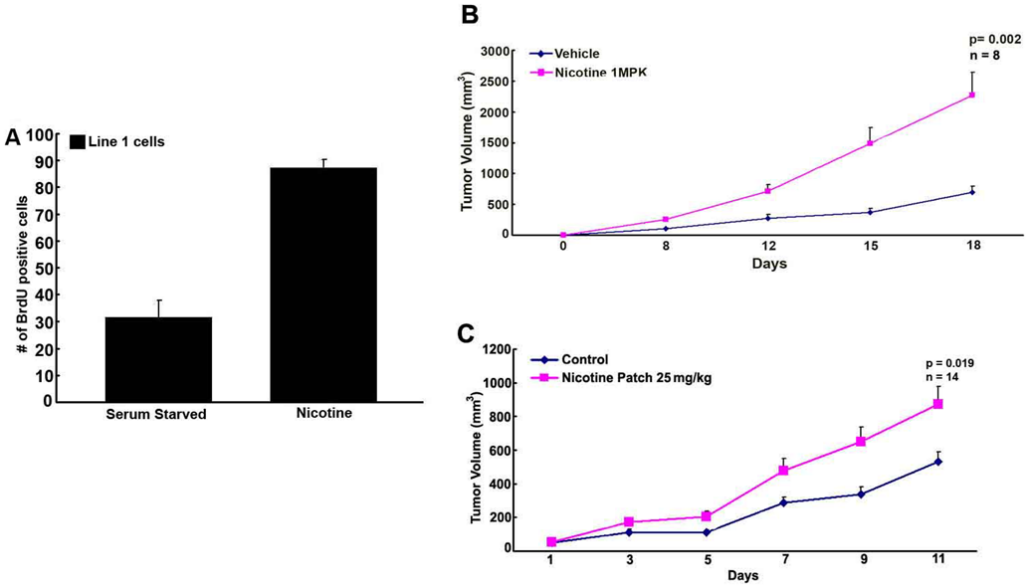
Nicotine promotes the growth of Line1 cells. (A) Nicotine (1 µM) promotes S-phase entry of serum starved Line1 cells in BrdU incorporation assays. (B) Nicotine (1 mg/kg) significantly increases Line1 tumor growth in Balb/c mice when administered by i.p. injection thrice weekly, p = 0.002, n = 10. (C) Nicotine (25 mg/kg/daily) also significantly increases Line1 tumor growth when administered by transdermal patches p = 0.019, n = 14.

The proliferative and pro-angiogenic effects of nicotine have been shown in multiple systems; recent studies had shown that nicotine could promote the growth of A549 (lung adenocarcinoma cells of type II alveolar phenotype) tumors in immunodeficient mice [25]. To determine the effects of nicotine on tumor growth and metastasis in immunocompetent mice, Line1 mouse adenocarcinoma cells were utilized. Line1 cells form subcutaneous (s.c.) tumors in BALB/c mice, which can metastasize to the lungs [34], [35], [36], [37], [38]. To examine whether nicotine induced proliferation of Line1 cells, the cells were serum starved for 72 hours and subsequently stimulated with 1 µM nicotine for 18 hours. S-phase entry was measured using BrdU incorporation assays. Nicotine could efficiently stimulate Line1 cells to enter S-phase (Figure 1A). Next, Line1 cells were implanted into the flanks of BALB/c mice and allowed to form tumors. The mice were randomized into two groups, with one group receiving vehicle (*n* = 8) and the second receiving 1 mg/kg nicotine (*n* = 8) thrice weekly by intraperitoneal (i.p.) injection. Mice that received nicotine had significantly larger tumors compared to those receiving vehicle; tumor volumes averaged 695±98 mm^3^ in vehicle treated mice, compared to 2267±369 mm^3^ in nicotine treated mice (Figure 1B), *p* = 0.002.

Based on the above results, experiments were done to examine whether nicotine administered by over-the-counter transdermal patches could promote tumor growth. BALB/c mice (*n* = 16) implanted with Line 1 tumors were randomized into two groups and nicotine patches were applied daily at a dose of 25 mg/kg nicotine. It was found that nicotine administered by transdermal patches could significantly increase the growth of Line1 tumors; control mice had an average tumor volume of 530±59 mm^3^ while mice wearing nicotine patches had an average volume of 871±106 mm^3^ (Figure 1C), *p* = 0.019. These experiments confirm that exposure to nicotine, even through nicotine supplements, might affect pre-established tumors.

### Nicotine promotes re-growth and metastasis of tumors in mice

**Figure 2:**
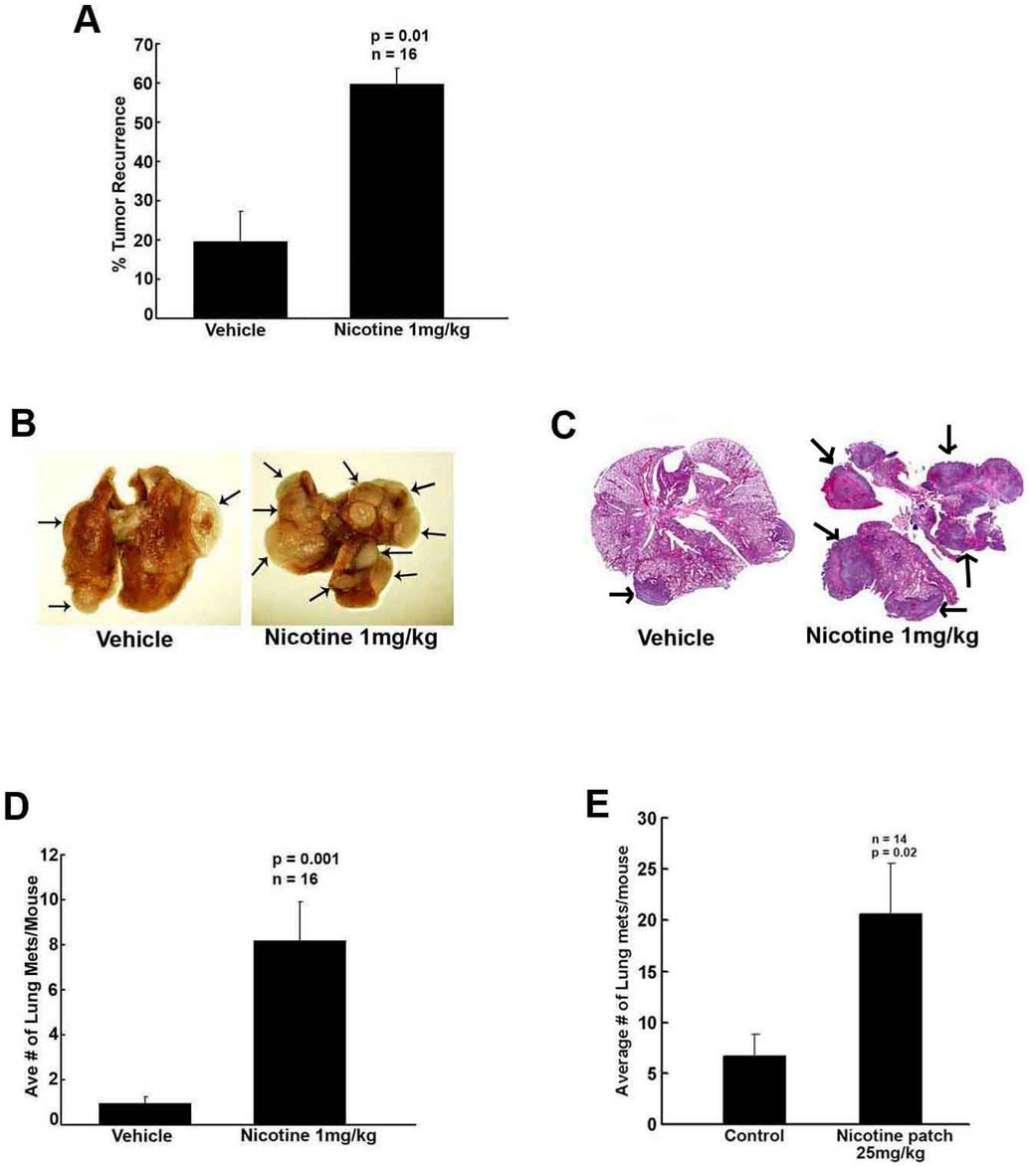
Nicotine increases metastatic potential. (A) Nicotine treated mice (1 mg/kg) displayed higher incidence of tumor recurrence following surgical removal of tumors compared to the vehicle control group, *p* = 0.01, *n* = 16. (B) Nicotine treated mice display significantly more lung metastasis from primary Line1 s.c. tumors. (C) H&E staining of lungs from vehicle and nicotine treated mice, nicotine treated mice display larger tumors as indicated by arrows. (D) Graph displaying the average total number of lung tumors per mouse in vehicle and nicotine (1 mg/kg) treated mice, p = 0.001, *n* = 16. (E) Graph displaying the average total number of lung tumors per mouse in control and nicotine patch (25 mg/kg) treated mice, p = 0.02, n = 16.

Since nicotine was found to enhance tumor growth, experiments were conducted to assess its effects on tumor metastasis. In order to examine this, the implanted tumors were surgically removed after 14 days of treatment or once they reached 500–700 mm^3^. Tumors were removed to prevent discomfort from large tumors. Mice were anesthetized for tumor removal, and wounds were stapled close. After the tumor removal, mice were administered vehicle or nicotine by i.p. injection for an additional 14 days. Interestingly, mice treated with nicotine showed a higher rate of tumor recurrence after the tumors were surgically removed (Figure 2A); vehicle treated mice displayed an average of 19±7% tumor recurrence, as compared to an average of 59±3% tumor recurrence in nicotine (1 mg/kg) treated mice, *p* = 0.01, *n* = 16. Tumor recurrence was calculated as percentage of recurring tumors out of the total number of tumors removed. As shown in Figure 2B, nicotine treated mice also displayed significantly greater number of lung metastases as well as larger metastatic foci compared to those receiving vehicle. Histologic examination of the lung tumors confirmed larger metastatic foci in the nicotine treated mice (Figure 2C). Mice receiving the vehicle had an average of 0.9±0.2 metastatic foci in the lungs per mouse; in comparison, mice that received nicotine at a dosage of 1 mg/kg thrice weekly had an average of 8.1±1.7 foci in the lungs per mouse, p = 0.001, *n* = 16 (Figure 2D). Interestingly, mice receiving nicotine through dermal patches had an average of 20.6±4.9 lung metastatic foci per mouse while mice receiving vehicle had an average of 6.7±2.1 foci per mouse (p = 0.02, n = 16) (Figure 2E).

Cotinine, a major metabolite of nicotine, is currently considered the best indicator of tobacco smoke exposure [39]. It is specific for nicotine, has a long half-life (15–40 hours), and its level is thought to be directly proportional to the quantity of absorbed nicotine [40]. Hence to determine the concentration of nicotine in our mouse models, we examined cotinine levels in the urine of the experimental mice. Urine (100 µl) was routinely collected throughout the length of the experiments. Mice receiving 1 mg/kg nicotine thrice weekly had an average cotinine concentration of 3000 ng/ml in urine. Mice that received 25 mg/kg nicotine by transdermal patch had an average cotinine concentration of 5000 ng/ml cotinine in their urine. Cotinine levels in urine were often in a wide range of concentration due to the variance of urine collection volumes. In human smokers, cotinine concentrations have been reported in values ranging from 1500 ng/ml to 8000 ng/ml [41], [42], [43], [44]. Nicotine doses used in these studies correlated well with cotinine levels in the urine of heavy smokers [42], [43].

### Nicotine enhances the growth of tumors induced by tobacco carcinogens

**Figure 3:**
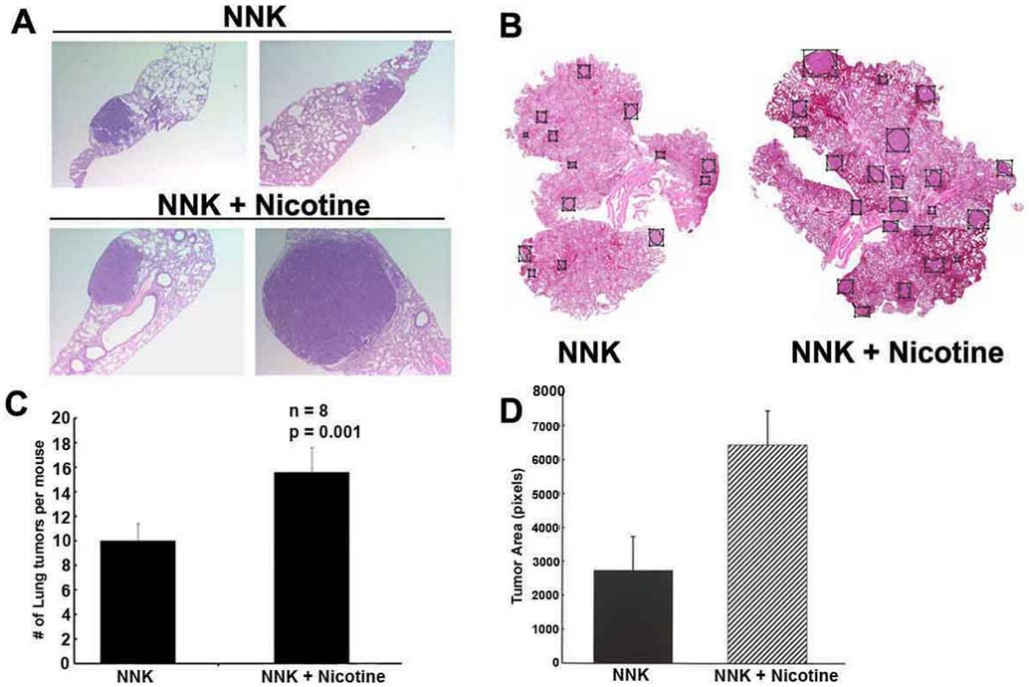
Nicotine increases number and size of NNK induced lung tumors. (A) H&E staining of transverse sectioning of lungs (B) Representative scanned images of H&E stained coronal lung sections, tumors are outlined by boxes. Images were scanned at 20×magnification. (C) Nicotine increases the average number of lung tumors per mouse, *p* = 0.01, *n* = 8. (D) Nicotine increased tumor size significantly in A/J mice.

Experiments were designed to examine the effects of nicotine on tumors induced by the tobacco carcinogen, NNK; this experimental system mimics a situation where tumors are initiated by a carcinogen, followed by exposure to nicotine alone. Towards this purpose, A/J mice were treated with 100 mg/kg NNK once a week for five weeks to initiate tumor formation and subsequently they were randomized into two groups. One group of mice received the vehicle (PBS) whilst the second group received nicotine (1 mg/kg) thrice weekly by i.p. injection; mice were treated with nicotine or vehicle for 28 weeks. Lungs from both vehicle and nicotine treated mice had developed tumors (Figure 3A & B). H&E stained lung sections were scanned and a pathologist (DC) outlined the tumor area; size and number of tumor foci were quantitated. Mice that received PBS after NNK injections had an average of 10±3.0 lung tumors per section and mice that received nicotine 1 mg/kg had 16±3.0 tumors per section, p = 0.01, n = 8 (Figure 3C). Tumor size (area) also increased in nicotine treated mice (Figure 3D). This suggests that exposure to nicotine once the tumors are already initiated can result in enhanced tumor growth.

### Nicotine induces cell proliferation via α_7_ nAChR subunit in mouse lung cancer

**Figure 4:**
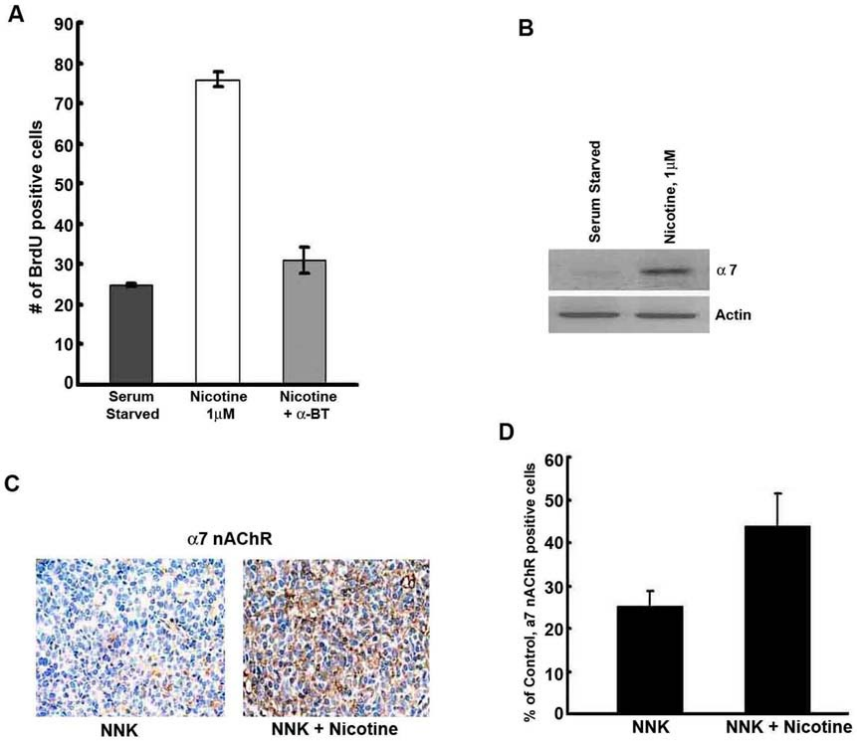
Nicotine enhances α_7_ nAChR subunit expression in Line1 cells. (A) Quiescent Line1 cells were treated with 1 µM nicotine for 18 h in the presence or absence of α-BT, an α_7_ nAChR subunit inhibitor. Nicotine enhances α_7_ expression, while α-BT reverses this. (B) Reverse-transcriptase coupled-PCR showing the expression of α_7_ nAChR subunits in serum starved Line1 cells treated with nicotine for 24 h. PCR for actin was used as the loading control. (C) α_7_ nAChR staining of A/J lung tumors induced by NNK or induced by NNK and exposed to Nicotine. Nicotine enhances the expression of this receptor subunit. (D) Quantitation of α_7_ nAChR expression in vehicle and nicotine treated tumors.

In our previous report, we showed that nicotine could induce proliferation and invasion of human NSCLCs via α_7_ nAChR subunit [45]. In the present study, we found nicotine could induce proliferation in mouse lung adenocarcinoma Line1 cells mainly via α_7_ nAChR subunit. α-bungarotoxin, an α_7_ subunit antagonist significantly inhibited nicotine-induced proliferation in Line1 cells, thereby suggesting that α_7_ subunit is mainly responsible for mediating the proliferative effect of nicotine (Figure 4A). To further confirm the role of α_7_ nAChR in nicotine mediated cell proliferation we performed RT-PCR for α_7_ nAChR subunit expression. Treatment of Line1 cells with nicotine for 24 hours caused the upregulation of α_7_ nAChR subunit with respect to serum-starved cells, as shown in Figure 4B. We observed a similar increase in the protein expression of α_7_ nAChR in lung tumor sections from A/J mice treated with nicotine upon immunostaining (Figure 4C); the results are quantified in Figure 4D. These results suggest that exposure to nicotine is inducing cell proliferation and tumor growth through the α_7_ subunit of nAChRs.

Nicotine has been shown to induce cell growth of colon and gastric cancer cells via α_7_ nAChR-mediated release of adrenaline, which in turn upregulates the expression of COX-2, PGE2 and VEGF, thereby facilitating the progression of these cancers [46], [47], [48]. Similarly, in our study we found that nicotine treatment elevated the levels of adrenaline and noradrenaline in mouse lung adenocarcinoma. Line1 cells were treated with nicotine for 48 hours and the media was collected to check for the release of adrenaline and noradrenaline. Nicotine elevated the levels of adrenaline to 42.5±2.4 pg/ml from 17±1 pg/ml in control, while noradrenaline levels peaked to 37±4.8 pg/ml when compared to 6.7±0.87 pg/ml in control. These results suggest that signaling through adrenaline might also contribute to the observed proliferative effects of nicotine.

### Nicotine facilitates EMT-like changes in lung cancers

**Figure 5:**
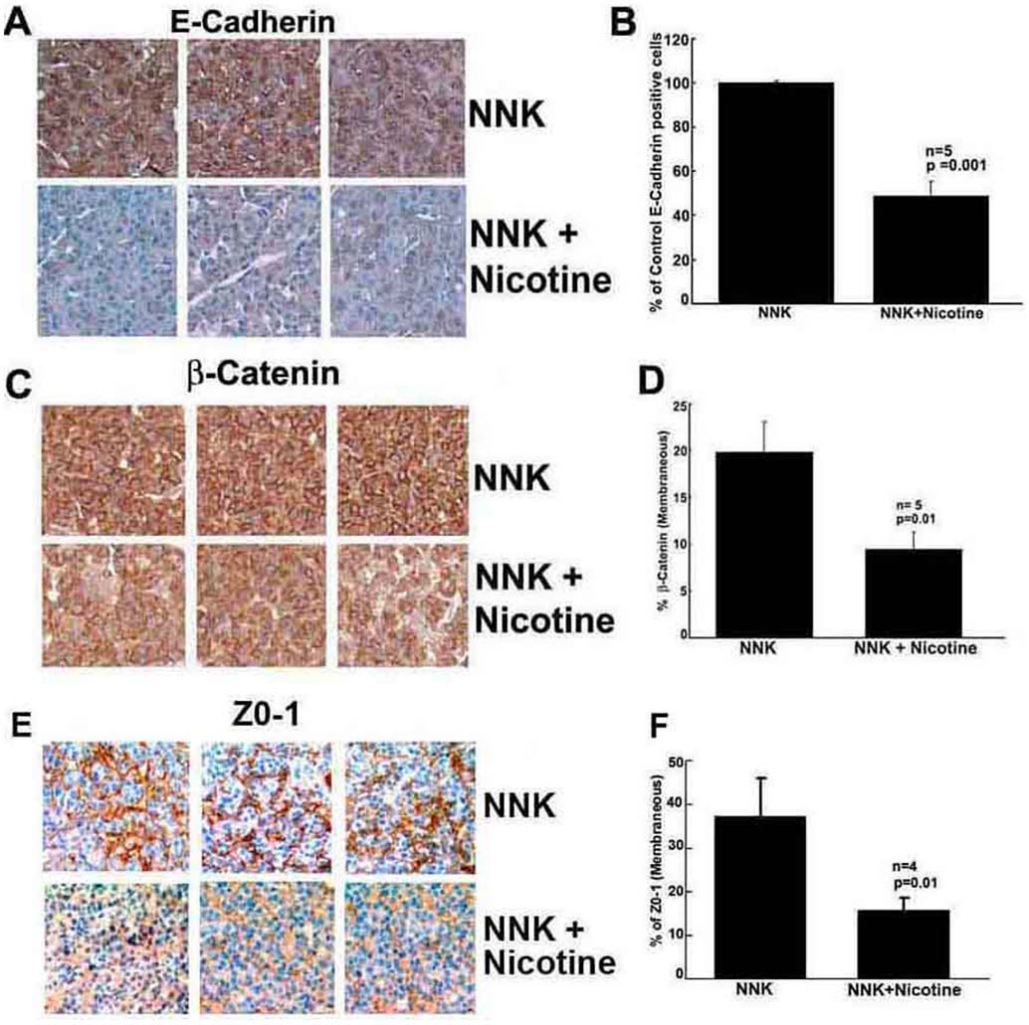
Nicotine reduced the expression of epithelial markers in A/J mice. (A) E-Cadherin staining of A/J lung tumors induced by NNK or NNK+nicotine. (B) Quantitation of E-Cadherin intensity in tumors (C) β-Catenin staining of A/J lung tumors induced by NNK or NNK+nicotine. (D) Quantitation of membranous β-Catenin. (E) ZO-1 staining of A/J lung tumors induced by NNK or NNK+nicotine. (F) Quantitation of membranous ZO-1.

Given the observation that nicotine can induce tumor growth and promote metastasis [20], [45], [49], attempts were made to understand the molecular events mediating these processes. Epithelial-mesenchymal transition (EMT) is a phenomenon by which cells lose their epithelial phenotype and acquire more mesenchymal features that facilitate detachment and migration [50], [51], [52]. We examined the tumors in A/J mice for changes consistent with an EMT-like phenomenon, using immunohistochemical staining for E-cadherin and β-Catenin, two proteins involved in the adhesion of epithelial cells. β-Catenin binds to E-Cadherin to facilitate cell adhesion and to exert its signaling functions. E-Cadherin levels were found to be significantly decreased in the tumors of mice treated with nicotine (Figure 5A); the results are quantified in Figure 5B. Staining for β-Catenin, which binds to E-Cadherin to facilitate adhesion in addition to its signaling functions [53], revealed loss of membranous localization of β-Catenin in lung tumors from mice treated with nicotine (Figure 5C & D). Moreover lung tumor sections from nicotine treated mice showed significantly reduced expression of ZO-1 upon immunostaining (Figure 5E & F). It was consistent with our earlier findings on breast and lung cancer cells that showed decreased membranous ZO-1 staining upon nicotine treatment, thereby indicating that nicotine facilitates the disruption of tight junctions to promote metastasis [45].

## Discussion

Several observations in patients suggest that those exposed to tobacco carcinogens are more likely to develop larger, more vascularized tumors with a high propensity for metastatic spread and resistance to chemotherapy [54]. In addition, about 30% of lung cancer patients who are smokers continue to smoke after they have been diagnosed [38]. This is problematic, as smokers who continue to use tobacco after a cancer diagnosis or return to smoking experience increased adverse medical consequences, such as: increased tumor progression, development of a second cancer, greater recurrence following successful treatment, greater cancer-related mortality and reduced quality of life [55], [56]. While these studies demonstrate a role for tobacco carcinogens in the initiation, growth and progression of cancers, the relative contribution of nicotine by itself to these processes is not known. This is a significant aspect, since the use of nicotine supplements is usually part of many cigarette smoking cessation programs.

While nicotine has been demonstrated to induce cell proliferation, angiogenesis and growth of tumors [20], the studies presented here show for the first time that nicotine patches can promote tumor re-growth and metastasis. Further, our results show that the presence of nicotine can enhance the growth of lung tumors initiated by a tobacco carcinogen. Essentially, the A/J mouse model is reflective of a situation where a smoker who has tumors initiated in the lung quits smoking and uses nicotine supplements to overcome the craving. Our results also show that a commercially available nicotine transdermal patch can promote the growth of tumors implanted into mice.

Our results as well as results from John Cooke's lab show that α7 receptor subunit is vital for nicotine-mediated cell proliferation and Src function is indispensable for nicotine to induce proliferation and angiogenesis [23], [24]. Another study has proposed the involvement of muscle-type nAChR subunits in proliferation [57]. Interestingly, three different studies from Europe showed a susceptibility locus for lung cancer that maps to nicotinic acetylcholine receptor subunit genes on chromosome15q24-25 [58], [59], [60]; this locus contained genes for α3, α5 and β4 subunits. Variations within this loci were predominantly found in smokers and correlated with smoking-related lung cancer as well as other diseases like peripheral arterial disease [61]. These recent studies raise the possibility that there is a direct correlation between the status and probably function of nAChRs and the onset as well as progression of lung cancer in smokers [62]. In addition, it has been shown that NSCLCs from never smokers express higher levels of α6β3 subunits whereas smokers show higher expression of α1, α3 and α7 subunits and a lower expression of α6β3 nAChR subunits [47]. Our studies presented in this manuscript suggest that nicotine stimulation of nAChR, and essentially activation of nAChR function, does indeed contribute to the progression of lung cancers.

There are many signaling molecules that are known to be activated by nAChR stimulation. These include activation of Src kinase cascade, PI3-Akt pathway, ERK/MAP kinase cascade, NFkB pathway as well as cyclic AMP signaling cascade. Further, nicotine has been shown to function in collaboration with estradiol [30]. These observations raise the possibility that a wide array of signals emanating from these receptors affect various aspects of tumor initiation, progression and metastatic spread. This scenario also opens up the possibility that targeting one or more of these pathways might be beneficial in combating such neoplasms.

The finding that epithelial adhesion molecules like E-Cadherin and its binding partner β-Catenin are affected by nicotine provides a molecular basis for these findings. It can be imagined that nicotine, through the nAChR signaling pathways, induces change in gene expression patterns to facilitate EMT and tumor metastasis. Indeed, it has been reported that the expression pattern of nAChR subunits is different in tumors from smokers and never smokers [63], [64]. Given the ability of nicotine to affect various aspects of tumor growth and metastasis, it is possible that antagonists of nAChR signaling might prove beneficial in controlling the growth and progression of lung cancers; certain studies support this contention [65]. Further, such agents that modulate the function of nAChRs such as varenidine, an agonist of α4β2 nAChRs, might be better alternatives for smoking cessation than nicotine itself.
