# Correction: Nicotine Promotes Tumor Growth and Metastasis in Mouse Models of Lung Cancer

**DOI:** 10.1371/journal.pone.0296477

**Published:** 2023-12-22

**Authors:** The PLOS ONE Editors

After publication of this article [1], concerns were raised about the second and third Z0-1 NNK+Nicotine panel in Fig 5E. Specifically, the left side of the middle NNK+Nicotine panel appears to overlap with a region on the right side of the rightmost NNK+Nicotine panel. The authors commented that this was due to an error made during the preparation of the figures and that whilst these panels represent the same experimental conditions, they are intended to be representative images of tissue taken from different mice.

The authors provided for editorial review replication data from the microscope slides used in the published article, including whole slide scans, representative images of regions of interest, and accompanying reanalysis via provision of individual datasets. These support the data presented in the published article and are provided in S1–S3 Files with this notice.

Whilst this information was provided by corresponding author Dr Srikumar Chellappan in 2015, PLOS has since been unable to contact Dr Chellappan to finalize the correction to this article. Therefore, the *PLOS ONE* editors issue this correction notice to share the replication data underlying Fig 5E with the permission of the corresponding author’s institute, The Moffitt Cancer Centre. We sincerely regret that this case was not resolved much sooner after the prior correspondence.

## Supporting information

S1 File Whole images of microscope slides taken from 4 mice per treatment group. Regions of interest are highlighted in green.
(ZIP)

S2 File Scans of a region of interest taken from the whole slide scans with 3 replicates per treatment group. (ZIP)

S3 File Individual level datasets produced from analysis of regions of interest. (ZIP)
