# Supplementary figures and images for: Correction: Nicotine Promotes Tumor Growth and Metastasis in Mouse Models of Lung Cancer
Source: PLoS One. 2023 Dec 22;18(12):e0296477. doi: 10.1371/journal.pone.0296477 (PMC10745137; doi:10.1371/journal.pone.0296477)

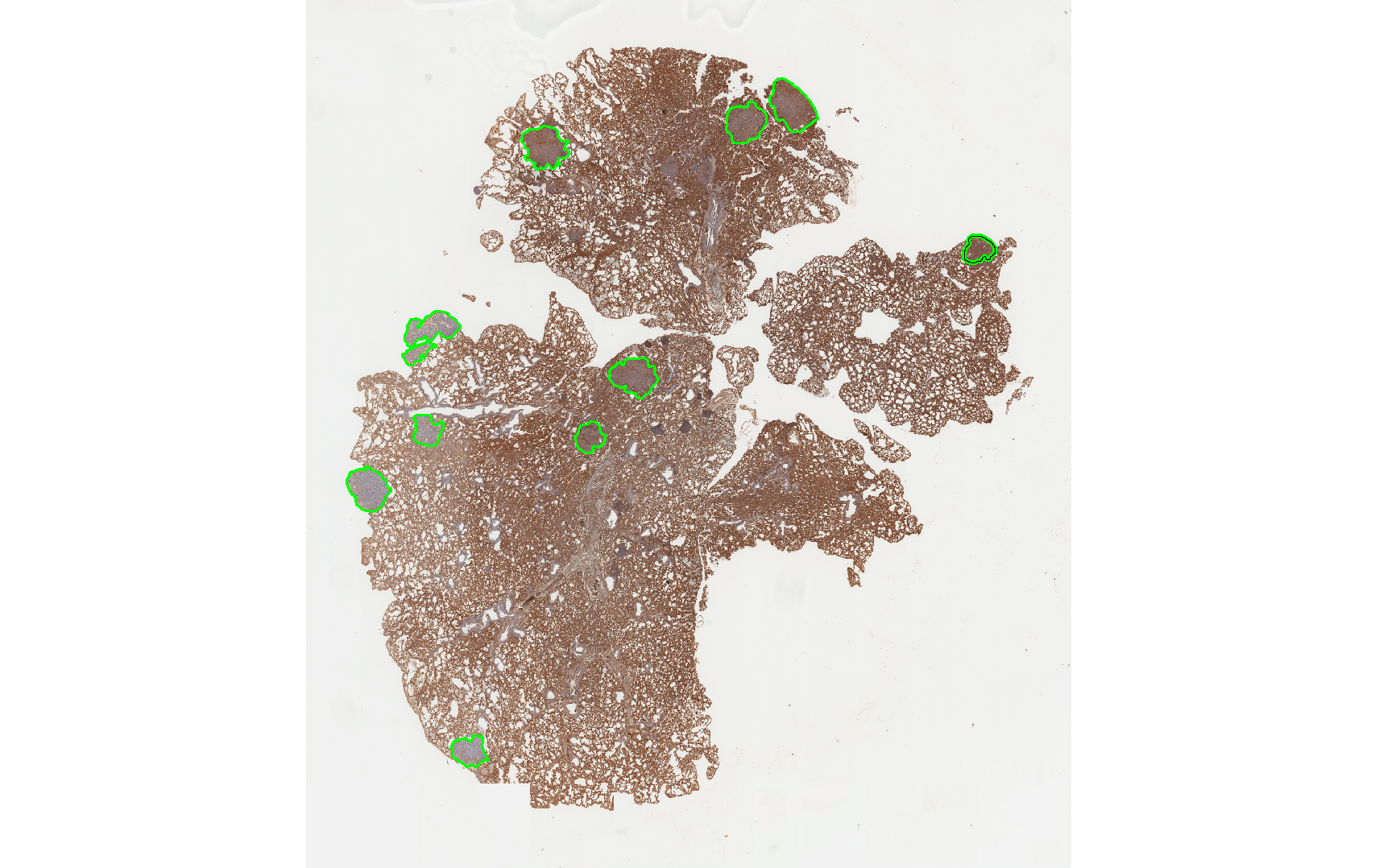

Supplement: S1 File — Regions of interest are highlighted in green. (ZIP) [file pone.0296477.s001.zip › Whole Slide Scans/NNK + Nic K5 whole with regions.tif]

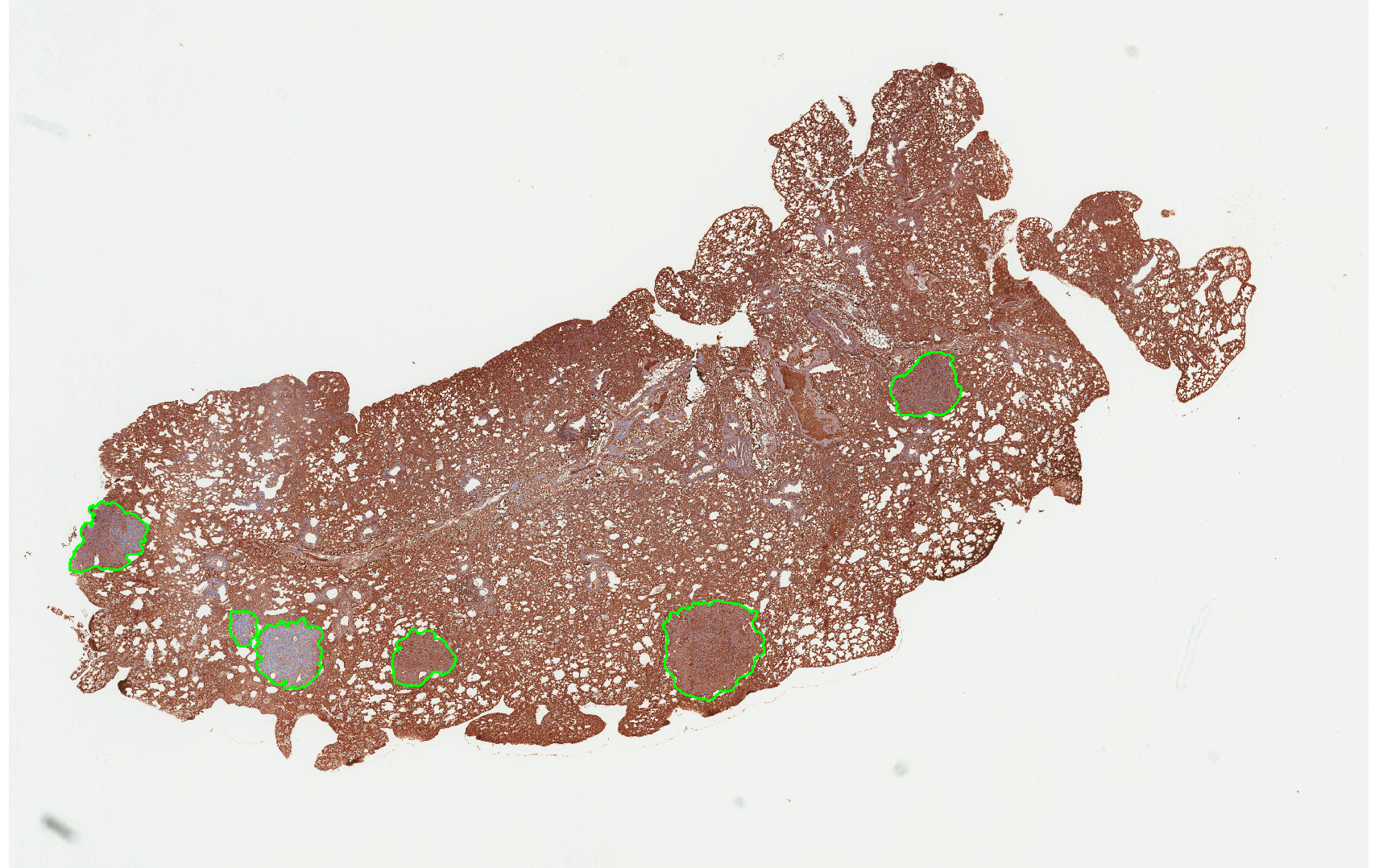

Supplement: S1 File — Regions of interest are highlighted in green. (ZIP) [file pone.0296477.s001.zip › Whole Slide Scans/NNK U5 whole with regions.tif]

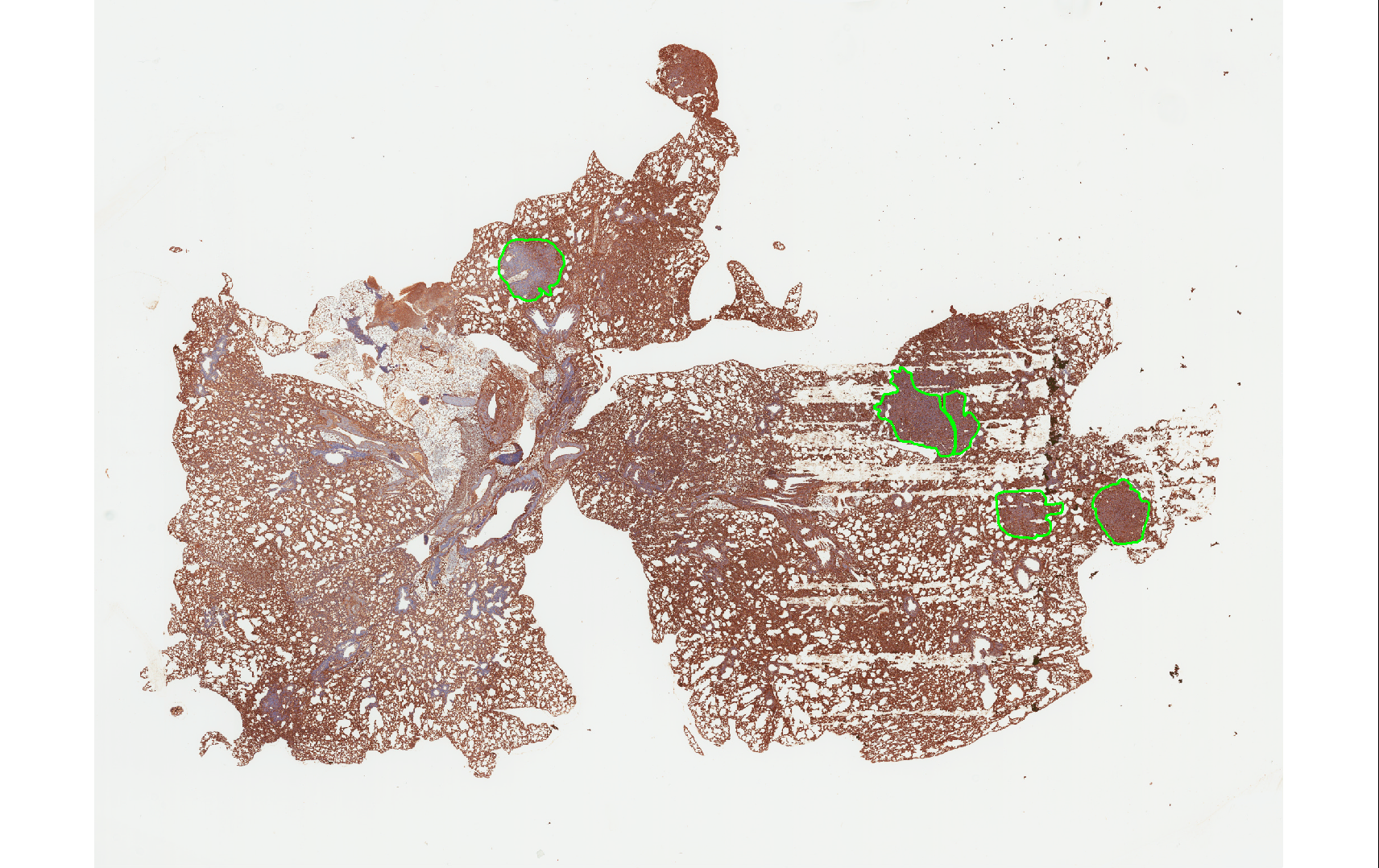

Supplement: S1 File — Regions of interest are highlighted in green. (ZIP) [file pone.0296477.s001.zip › Whole Slide Scans/NNK Q5 whole with regions.tif]

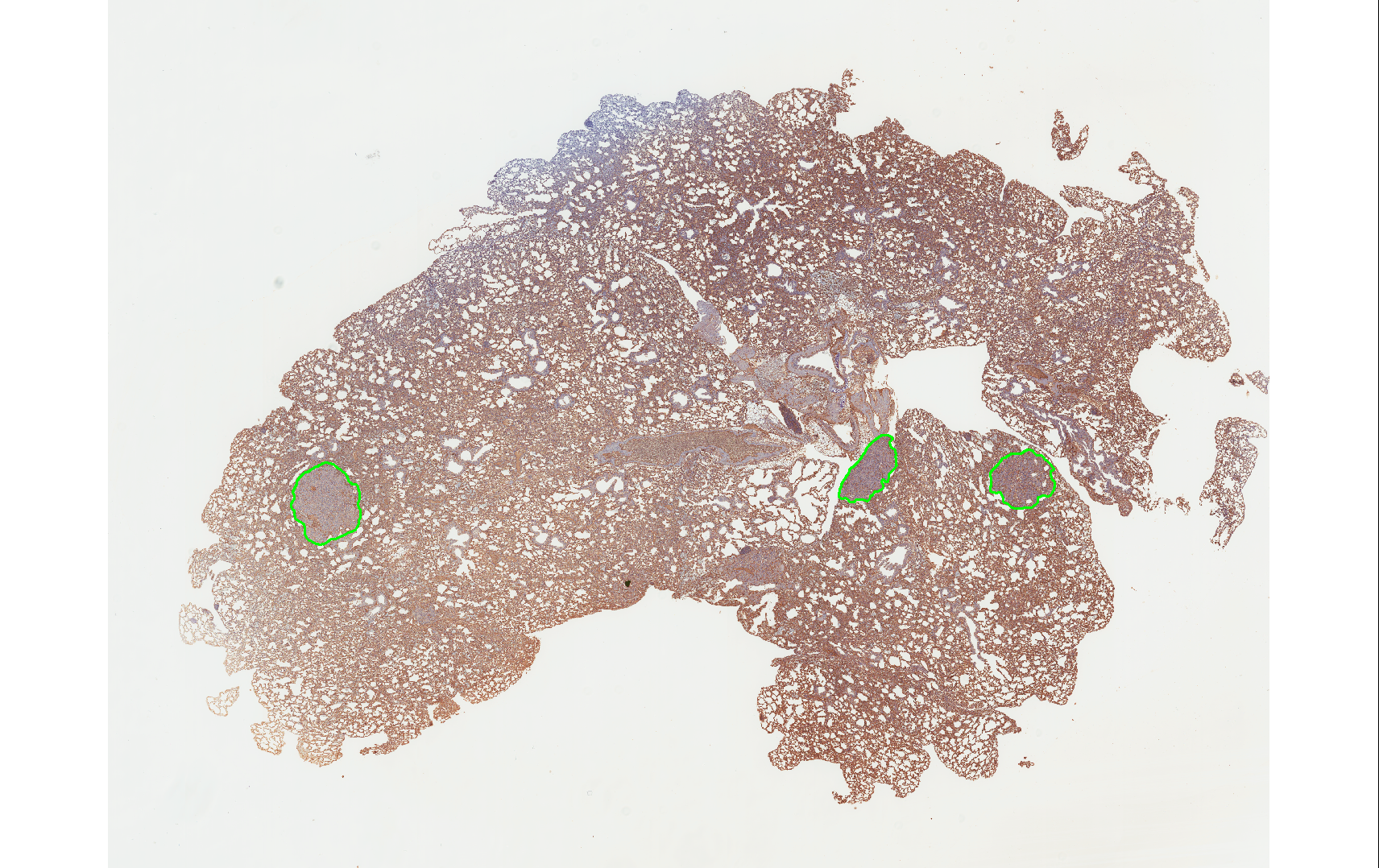

Supplement: S1 File — Regions of interest are highlighted in green. (ZIP) [file pone.0296477.s001.zip › Whole Slide Scans/NNK + Nic A6 whole with regions.tif]

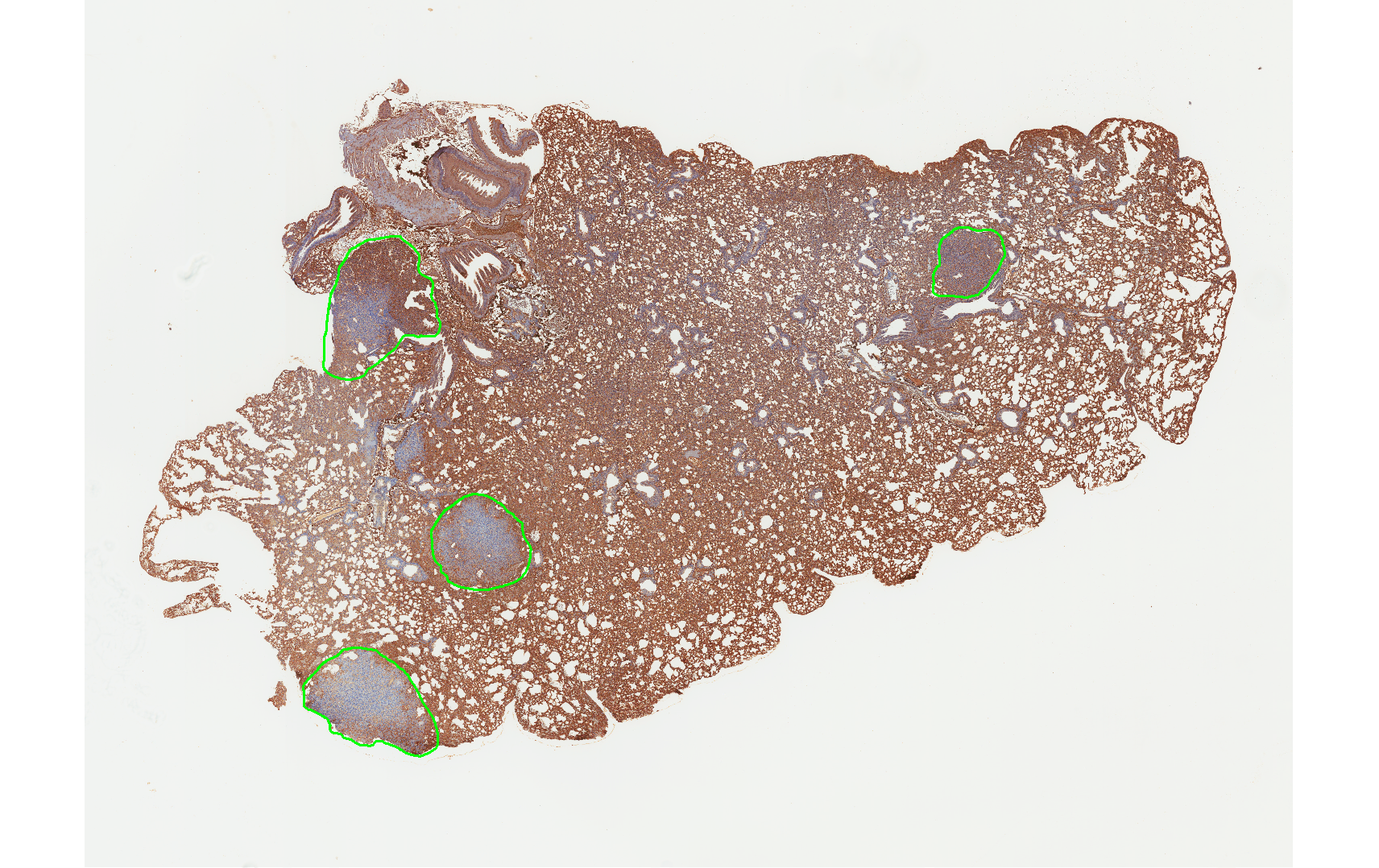

Supplement: S1 File — Regions of interest are highlighted in green. (ZIP) [file pone.0296477.s001.zip › Whole Slide Scans/NNK O5 whole with regions.tif]

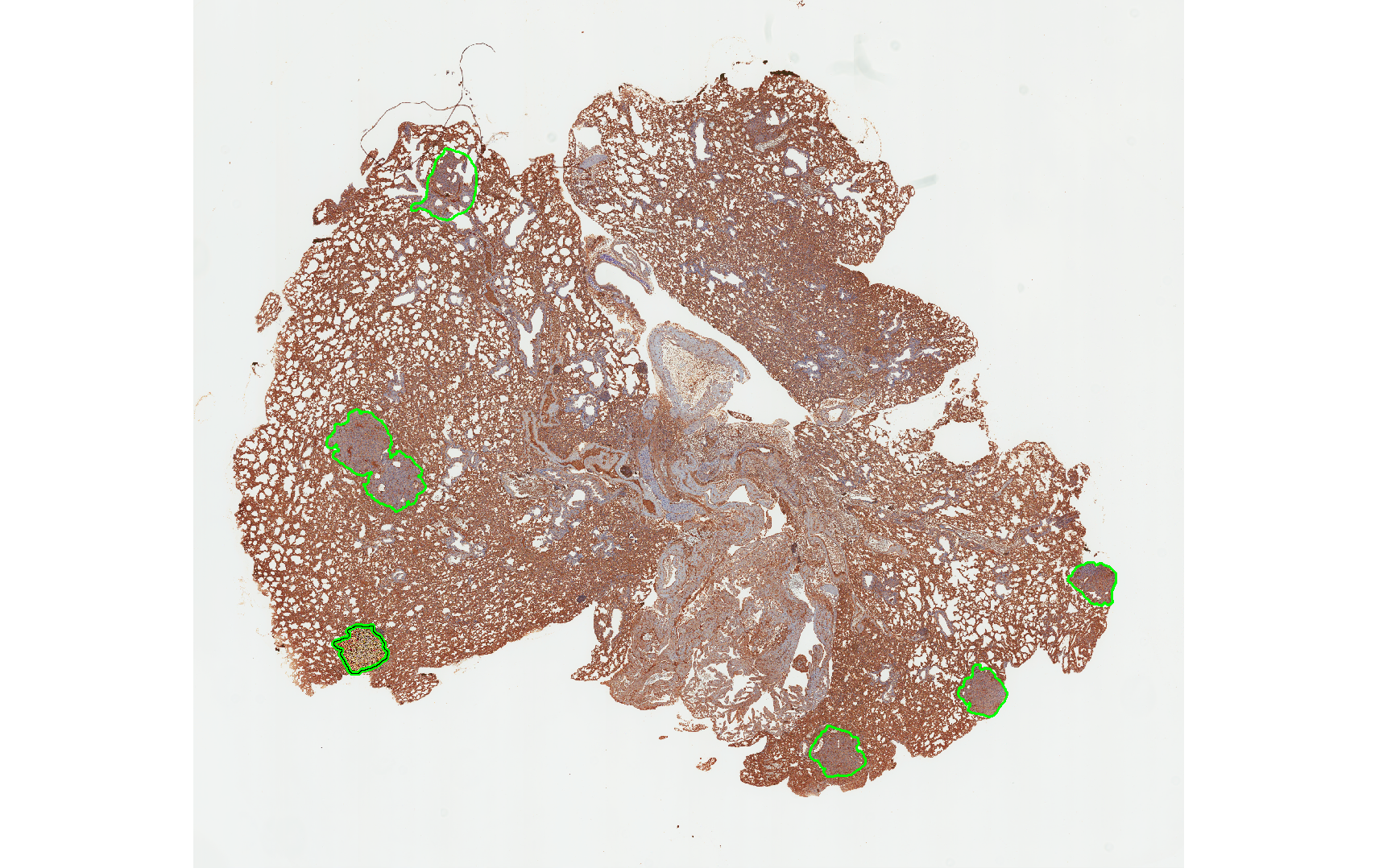

Supplement: S1 File — Regions of interest are highlighted in green. (ZIP) [file pone.0296477.s001.zip › Whole Slide Scans/NNK + Nic L5 whole with regions.tif]

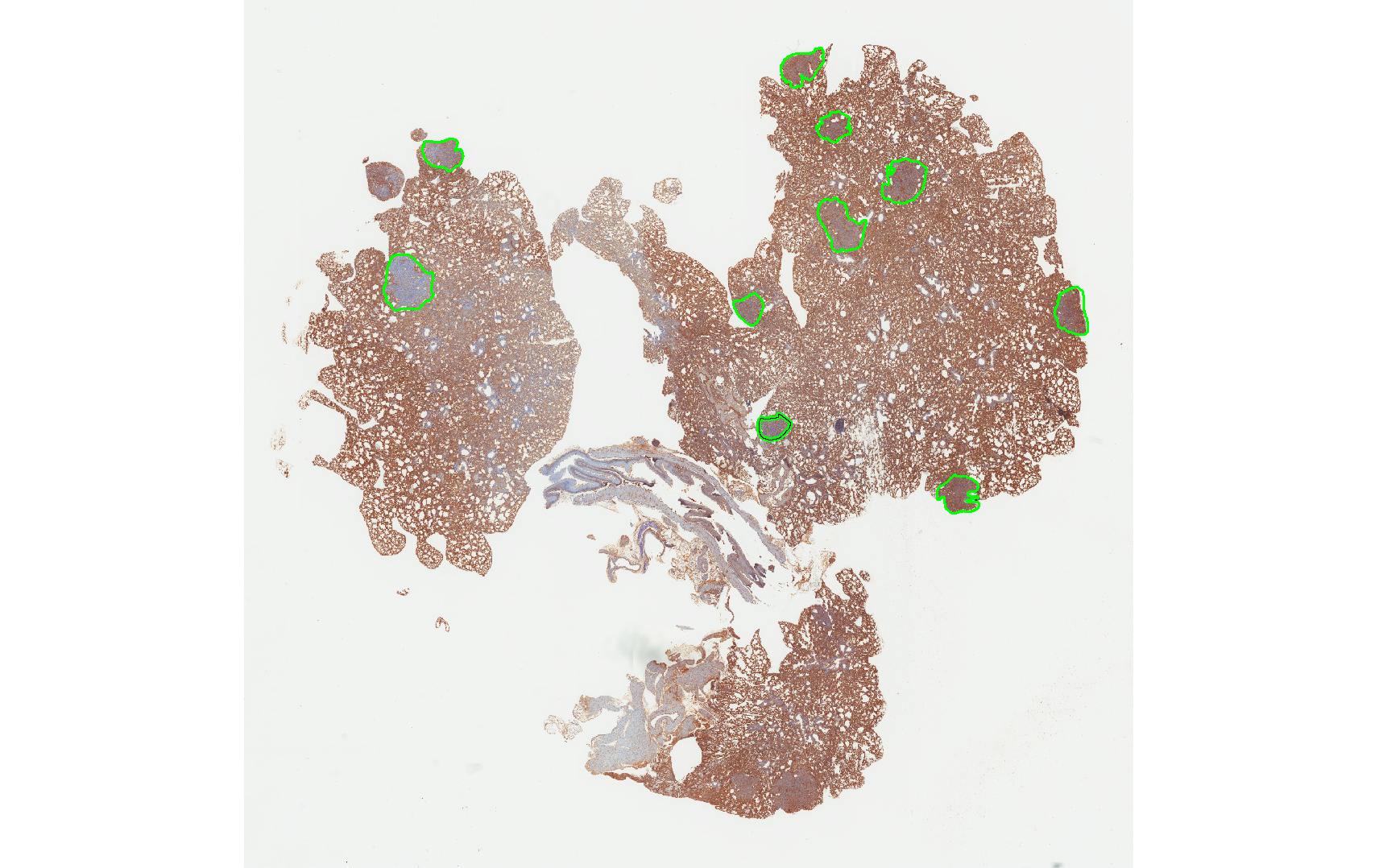

Supplement: S1 File — Regions of interest are highlighted in green. (ZIP) [file pone.0296477.s001.zip › Whole Slide Scans/NNK J5 whole with regions.tif]

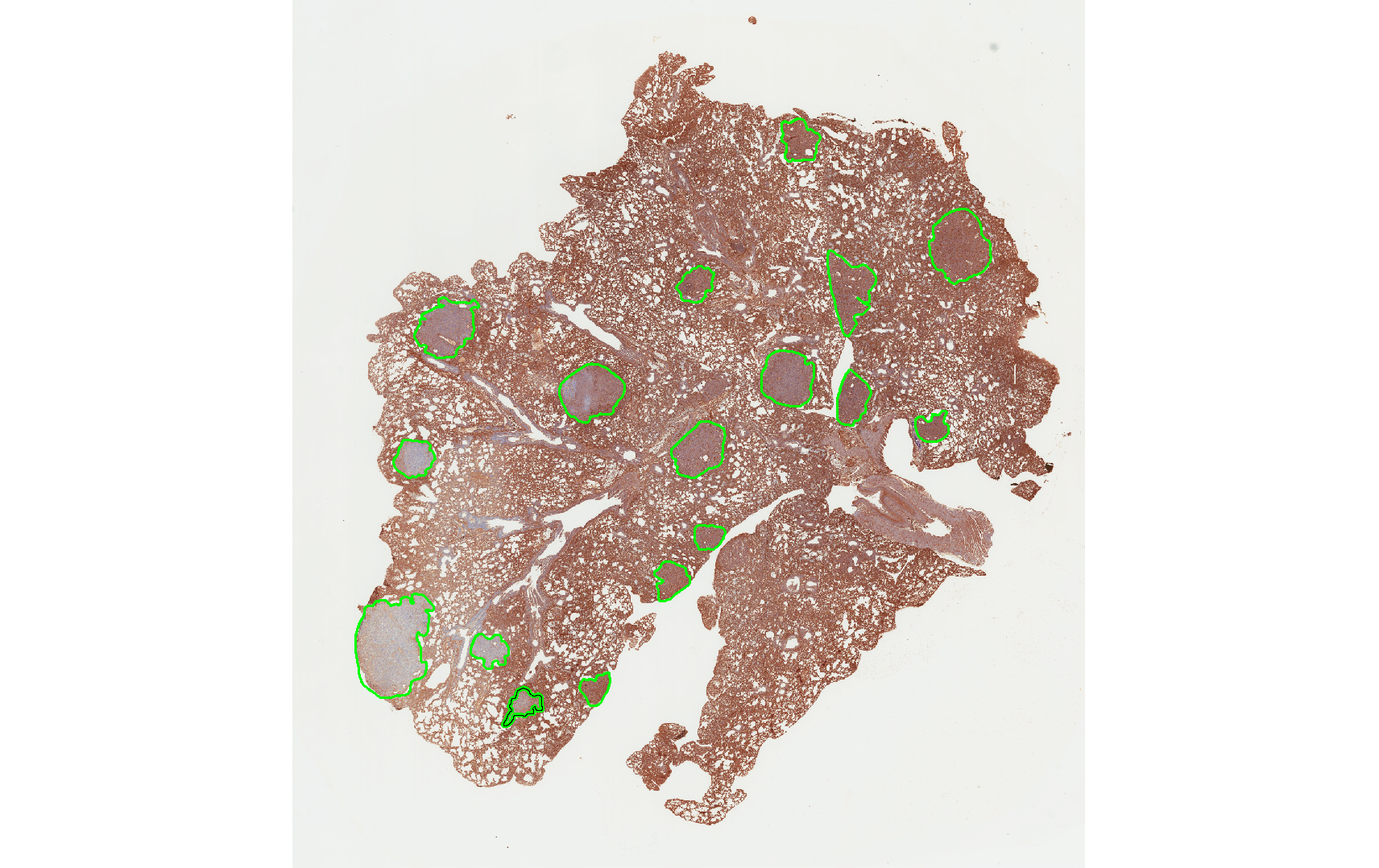

Supplement: S1 File — Regions of interest are highlighted in green. (ZIP) [file pone.0296477.s001.zip › Whole Slide Scans/NNK + Nic W5 whole with regions.tif]

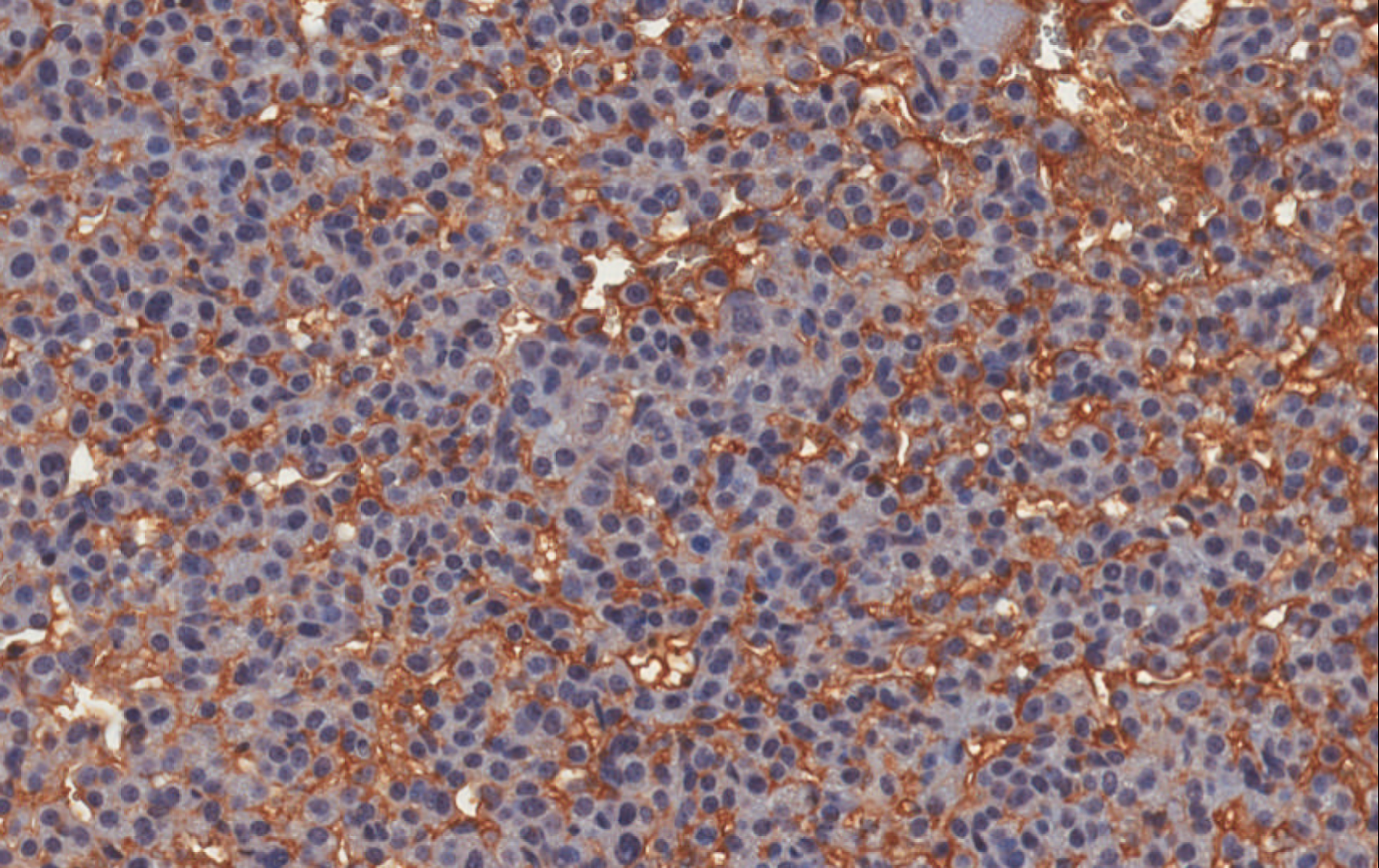

Supplement: S2 File — (ZIP) [file pone.0296477.s002.zip › NNK O5-1.tif]

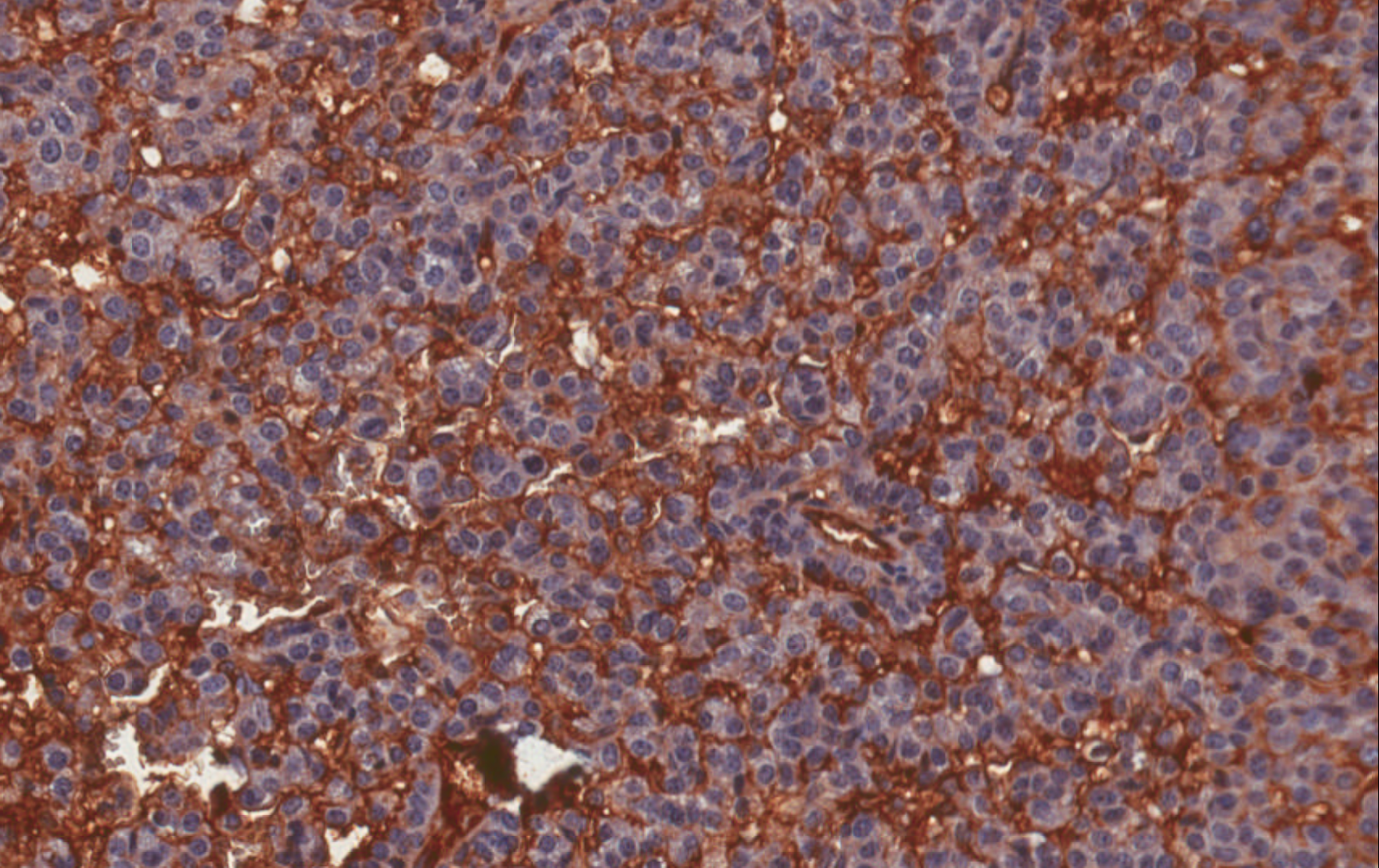

Supplement: S2 File — (ZIP) [file pone.0296477.s002.zip › NNK Q5-1.tif]

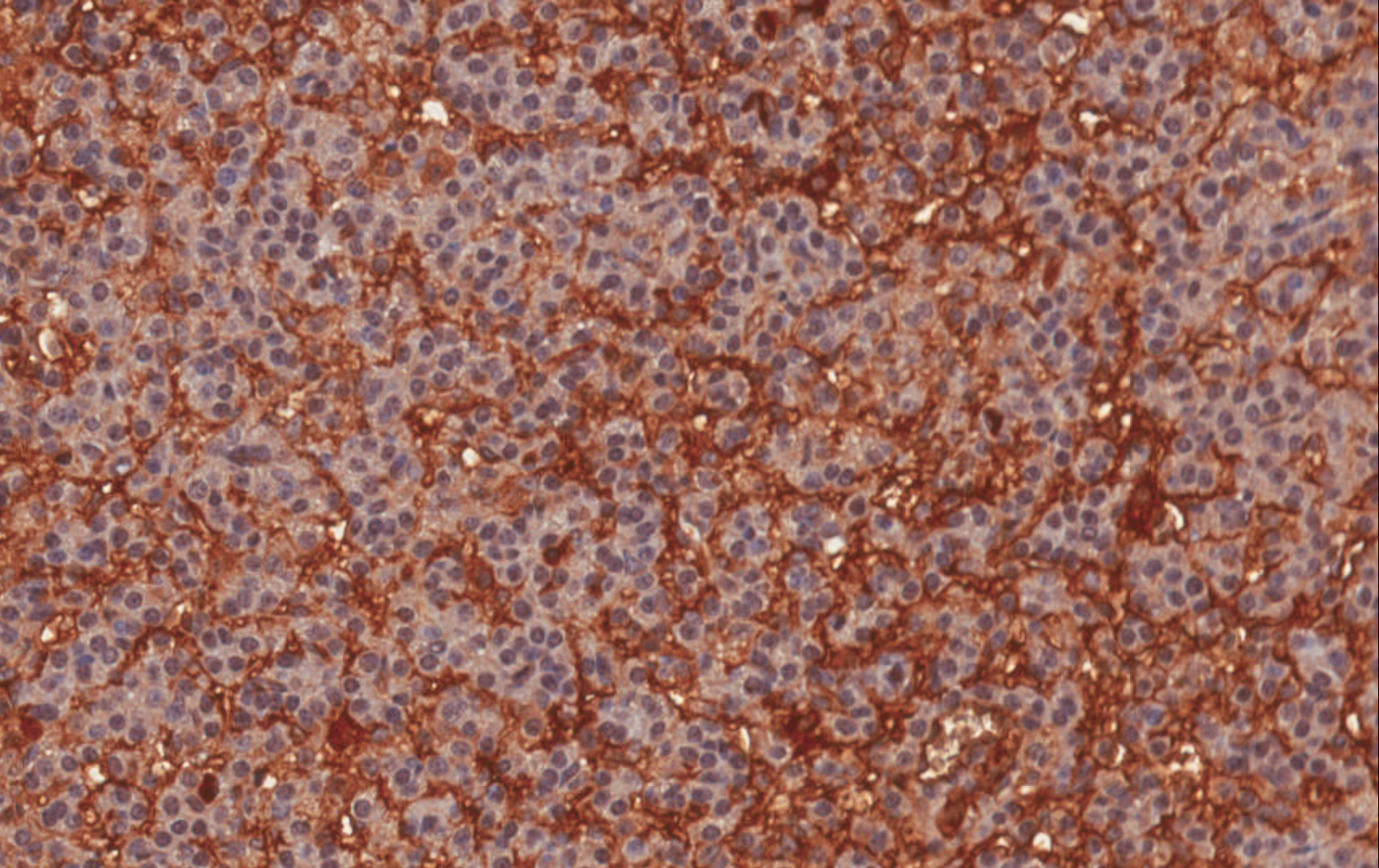

Supplement: S2 File — (ZIP) [file pone.0296477.s002.zip › NNK U5-1.tif]

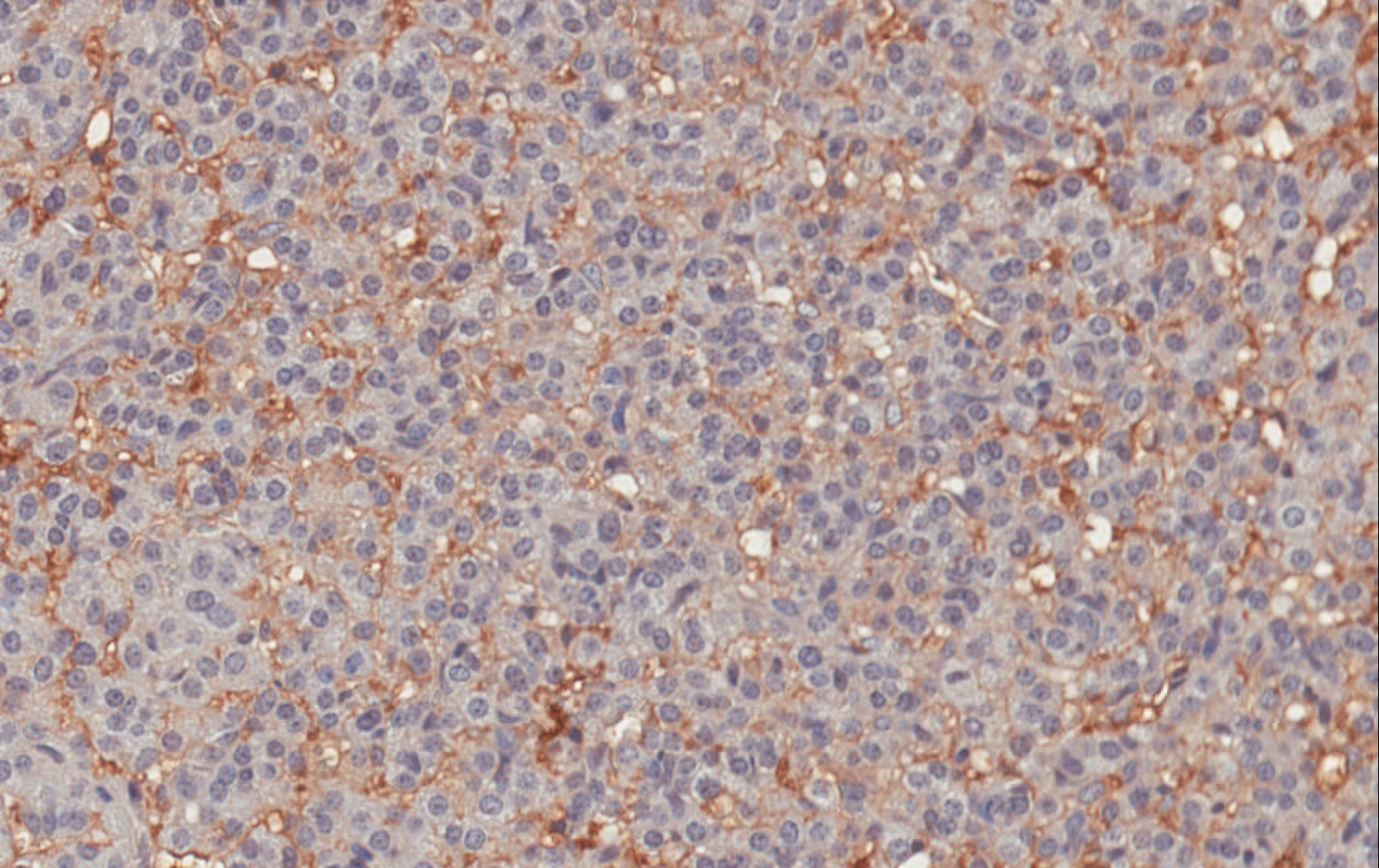

Supplement: S2 File — (ZIP) [file pone.0296477.s002.zip › NNK + Nic A6-1.tif]

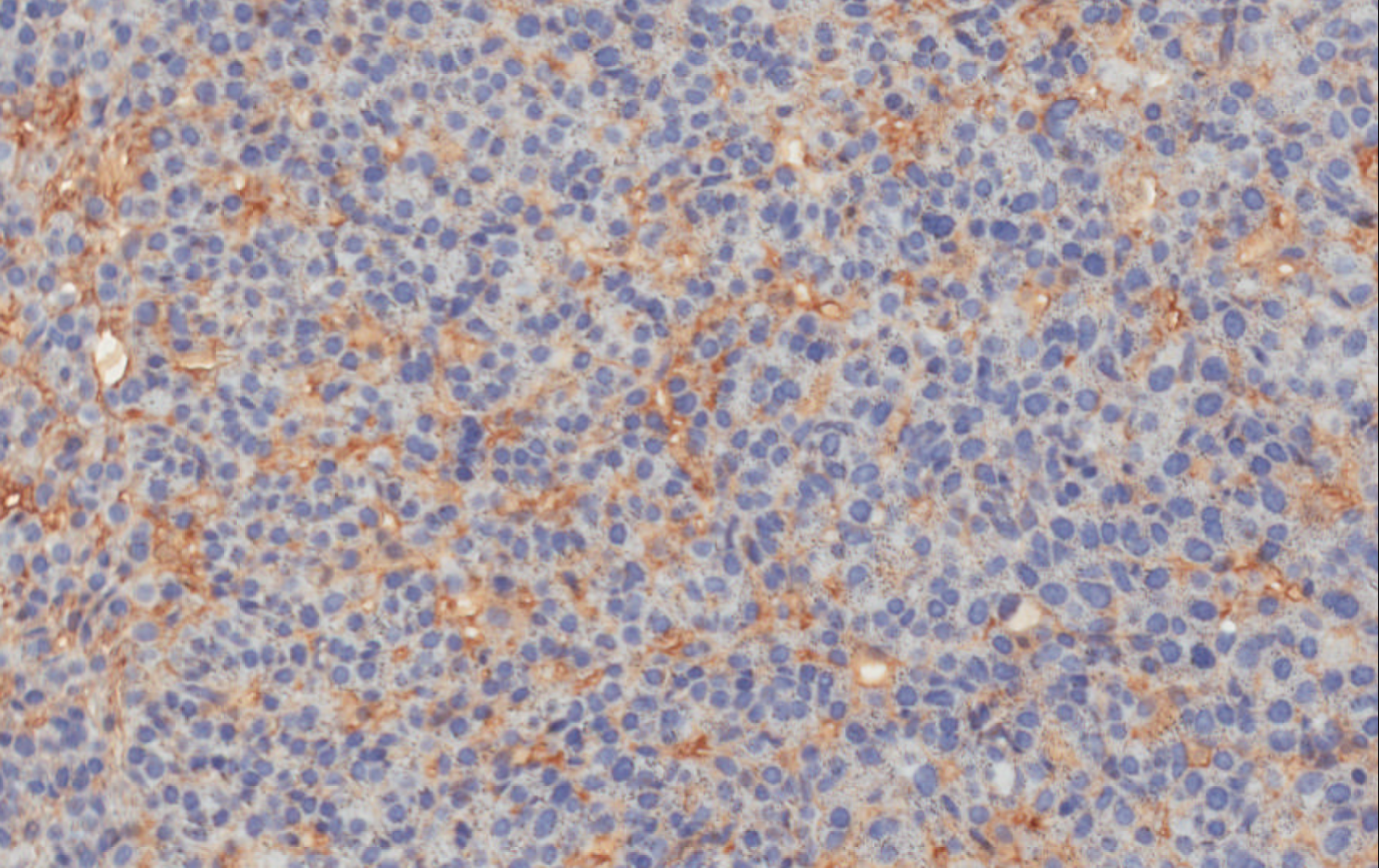

Supplement: S2 File — (ZIP) [file pone.0296477.s002.zip › NNK + Nic K5-1.tif]

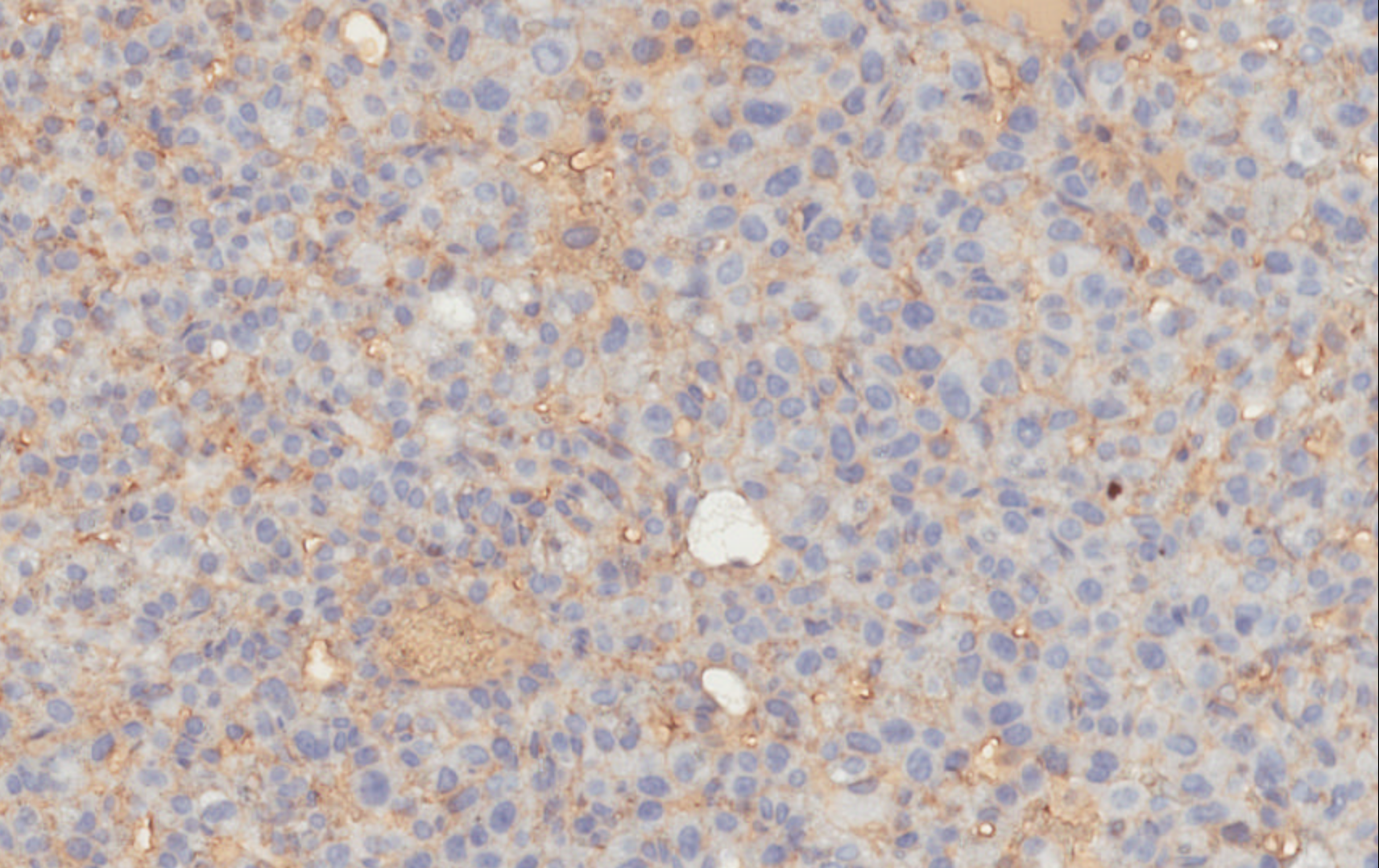

Supplement: S2 File — (ZIP) [file pone.0296477.s002.zip › NNK + Nic W5-2.tif]
